# Development of DNA Microarray for Indication of Viral Community-Acquired Pneumonia Pathogens

**DOI:** 10.17691/stm2025.17.3.02

**Published:** 2025-06-30

**Authors:** N.A. Sakharnov, E.N. Filatova, M.I. Popkova, L.B. Lukovnikova, M.O. Bahmeteva, S.L. Slavin, O.V. Utkin

**Affiliations:** Senior Researcher, Laboratory of Molecular Biology and Biotechnology; Academician I.N. Blokhina Nizhny Novgorod Scientific Research Institute of Epidemiology and Microbiology of Rospotrebnadzor (Russian Federal Consumer Rights Protection and Human Health Control Service), 71 Malaya Yamskaya St., Nizhny Novgorod, 603950, Russia; Leading Researcher, Laboratory of Molecular Biology and Biotechnology; Academician I.N. Blokhina Nizhny Novgorod Scientific Research Institute of Epidemiology and Microbiology of Rospotrebnadzor (Russian Federal Consumer Rights Protection and Human Health Control Service), 71 Malaya Yamskaya St., Nizhny Novgorod, 603950, Russia; Leading Researcher, Laboratory of Molecular Biology and Biotechnology; Academician I.N. Blokhina Nizhny Novgorod Scientific Research Institute of Epidemiology and Microbiology of Rospotrebnadzor (Russian Federal Consumer Rights Protection and Human Health Control Service), 71 Malaya Yamskaya St., Nizhny Novgorod, 603950, Russia; Associate Professor, Department of Molecular Biology and Immunology; National Research Lobachevsky State University of Nizhny Novgorod, 23 Prospekt Gagarina, Nizhny Novgorod, 603022, Russia; Student; National Research Lobachevsky State University of Nizhny Novgorod, 23 Prospekt Gagarina, Nizhny Novgorod, 603022, Russia; Student; National Research Lobachevsky State University of Nizhny Novgorod, 23 Prospekt Gagarina, Nizhny Novgorod, 603022, Russia; Head of the Laboratory of Molecular Biology and Biotechnology; Academician I.N. Blokhina Nizhny Novgorod Scientific Research Institute of Epidemiology and Microbiology of Rospotrebnadzor (Russian Federal Consumer Rights Protection and Human Health Control Service), 71 Malaya Yamskaya St., Nizhny Novgorod, 603950, Russia

**Keywords:** DNA microarray, community-acquired pneumonia, adenovirus, bocavirus, viral pathogens, respiratory syncytial virus, rhinovirus, coronavirus SARS-CoV-2

## Abstract

**The aim of the study** is to develop a DNA microarray for the indication of viral pathogens causing community-acquired pneumonia.

**Materials and Methods:**

The study materials were swab samples from the nasopharyngeal and oropharyngeal mucous membranes of patients aged 2 months to 18 years with X-ray confirmed pneumonia. The selection of DNA probes for the specific detection of viral community-acquired pneumonia pathogens and development of the microarray design were carried out using our previously developed disprose program. The nucleotide sequences of pathogens were obtained from the NCBI Nucleotide and GISAID databases. The selected DNA probes were synthesized on CustomArray slides (USA). The optimal hybridization temperature was selected on a model pooled sample containing adenovirus DNA and SARS-CoV-2 coronavirus RNA. The selection criteria were the percentage of effective probes with a standardized hybridization signal (SHS) ≥3 Z and the excess of SHS levels of effective specific probes compared to SHS of effective non-specific probes. The DNA probes were selected for the specific detection of viral community-acquired pneumonia pathogens, characterized by an effective hybridization signal under the identified conditions. Using ROC analysis, threshold values of specific probe signals were established, the excess of which was interpreted as the evidence of the pathogen presence in a sample.

**Results:**

A microarray design included 544 DNA probes for the detection of adenovirus, bocavirus, respiratory syncytial virus, metapneumovirus, parainfluenza virus, rhinovirus, and coronavirus. The DNA probes were synthesized on slides. The optimal DNA hybridization temperature on microarrays was established (47°C). A list of probes for specific detection of adenovirus group B, bocavirus, parainfluenza virus type 3, respiratory syncytial virus, rhinovirus, and SARS-CoV-2 coronavirus, characterized by an effective hybridization signal under the identified conditions, was selected. The threshold values of probe signals for specific detection of these pathogens in clinical samples were determined.

**Conclusion:**

A DNA microarray for the indication of viral community-acquired pneumonia pathogens was developed and synthesized. The interpretation of the hybridization results corresponds to the results obtained by the PCR method. The developed microarray can be used to improve laboratory diagnostics of viral community-acquired pneumonia pathogens.

## Introduction

Community-acquired pneumonia (CAP) is an acute infectious lung inflammation that develops outside of a hospital setting or is diagnosed within the first two days of hospitalization [1]. CAP remains a significant public health issue due to its high incidence rates. In 2023, the CAP incidence in Russia was 498.02 cases per 100,000 population; with rates of 803.6 per 100,000 among children and of 1465.5 per 100,000 among those aged 1–2 years [2]. The CAP etiological agents frequently include respiratory viruses such as human metapneumovirus (HMPV), respiratory syncytial virus (RSV), rhinovirus (RV), adenovirus (HAdV), human parainfluenza virus (HPIV), and coronaviruses (HCoV) [3–6]. The identification of a CAP etiological agent is crucially necessary for improving the treatment effectiveness; however, the CAP pathogens are often detected in individuals without clinical signs of the disease. The identification of multiple potential CAP pathogens may indicate healthy carriage, asymptomatic infection, or persistence following infection [7]. Quantitative PCR analysis is one of the reliable methods for identifying the predominant pathogen that is presumed to cause the CAP syndrome [8]. Given the constantly expanding spectrum of CAP pathogens, the implementation of PCR tests for the detection of each of them is a labor-intensive and costly procedure.

DNA microarrays enable multiplex detection of various pathogens, achieving an optimal balance of performance, accuracy, and cost [9]. Internationally, DNA microarrays have been developed to detect a wide range of bacteria and viruses, including pathogens responsible for respiratory conditions. For instance, the LLMDA (Lawrence Livermore Microbial Detection Array; Lawrence Livermore National Laboratory, USA) can detect 2200 viral species and 900 bacterial species [10, 11], while the Axiom Microbiome Array (Axiom, USA) can identify 6091 bacterial species and 4770 viral species [12]. However, the range of pathogens detected by these DNA microarrays exceeds what is necessary for the identification of clinically significant pathogens. Additionally, their application is hindered by high costs and the complexity of sample preparation process, which involves the use of numerous specific primers and multiple reaction mixtures. Genomica (Spain) has developed low-density DNA microarrays, CLART, for the detection of the most common viral CAP pathogens (these microarrays have undergone clinical trials). They are unable to differentiate between colonization and an active infection [13]; therefore, the DNA microarrays development for the viral CAP pathogens detection is relevant. The advantage of such microarrays would be the use of random primers, simplifying and reducing the cost of the sample preparation process while also facilitating the differential diagnosis of active infection and pathogen carriage.

**The aim of the study** is to develop a DNA microarray for the indication of viral pathogens causing community- acquired pneumonia.

## Materials and Methods

### DNA probe selection and synthesis

Using our own algorithm disprose [14], DNA probes were selected for the diagnosis of adenovirus groups B, C, and E; bocavirus (HBoV); respiratory syncytial virus; metapneumovirus; human parainfluenza viruses types 1–4; rhinovirus; coronavirus species HKU-1, OC-43, NL-63, E-229, and SARS-CoV-2. Nucleotide sequences of the CAP pathogens were obtained from the NCBI Nucleotide and GISAID databases [15, 16]. A total of 544 target DNA probes, each 25–30 nucleotides in length, were selected and subsequently synthesized on CustomArray Blank Slide 12K (CustomArray, USA). The slide contained four identical sectors, each comprising three blocks of 544 target DNA probes, three blocks of 30 DNA probes specific to the *Rhizobium rubi* genome used as a negative control, and three blocks of 30 quality control probes for synthesis. The synthesis of probes was conducted according to the manufacturer’s protocol using the B3 Synthesizer (CustomArray, USA) and reagent kits from Merck Sharp & Dohme (USA), Sigma (USA, Germany, France), Panreac (Spain), and Biohim (Russia).

### Materials

The material consisted of swabs from the nasopharyngeal and oropharyngeal mucous membranes of patients aged 2 months to 18 years with X-ray confirmed pneumonia. In accordance with the Declaration of Helsinki (2024), informed consent was obtained from patients and representatives of under- legal-age patients by doctors of healthcare institutions.

### Sample selection for the research

The presence of CAP pathogens was performed using commercial kits AmpliSens ORVI-Screen-FL and AmpliSens CoV- Bat-FL (Central Research Institute of Epidemiology of Rospotrebnadzor, Russia). Clinical samples used in the study contained, according to PCR testing results, exclusively DNA/RNA-containing pathogens: adenovirus (10 units), bocavirus (8 units), SARS-CoV-2 coronavirus (12 units), human parainfluenza virus type 3 (10 units), respiratory syncytial virus (12 units), and rhinovirus (12 units). A pooled sample of swabs from the nasopharyngeal and oropharyngeal mucous membranes from healthy donors with negative PCR results for CAP pathogens served as the negative control sample.

### Nucleic acid sample preparation protocol for hybridization

The total nucleic acid (NA) was extracted from the samples using a RIBO-prep commercial kit (Central Research Institute of Epidemiology of Rospotrebnadzor, Russia) and further purified by isopropanol precipitation (Biohim, Russia). The obtained NA was divided into two portions: one was used for the sample preparation of DNA-containing pathogens (HAdV, HBoV), while the other was utilized for RNA- containing pathogens (HPIV, HRSV, RV, HMPV, SARS- CoV-2).

The sample preparation process for DNA-containing pathogens involved several stages. Total NA was fragmented using the NEBNext dsDNA Fragmentase kit (New England Biolabs, Great Britain). DNA was precipitated with isopropanol and 3 M sodium acetate (pH 7.0). DNA amplification was performed using random primers (Random (dN)10 primer; Evrogen, Russia) and a reagent kit (Encyclo Plus PCR kit; Evrogen, Russia) using an MaxyGene Gradient thermal cycler (Axygen, USA). The reaction temperature profile was the following: 95°C for 2 min; 25 cycles (95°C for 30 s, 30°C for 30 s, 72°C for 6 min); 72°C for 8 min. The obtained DNA was precipitated with isopropanol and 3 M sodium acetate (pH 7.0) and used for *in vitro* transcription with random primers (Random (dN)10 primer; Evrogen, Russia) and the DNA Polymerase 1 *E. coli* (Klenow fragment) reagent kit (SibEnzyme, Russia). Half of the deoxyuridine triphosphate (dUTP) was replaced with its biotinylated modification, Bio- 12-dUTP (DNA-Synthesis, Russia). The biotin-labeled DNA was precipitated with isopropanol and 3 M sodium acetate (pH 7.0).

During the sample preparation of total NA with RNA- containing pathogens, a reverse transcription stage was added to the standard procedure using the MINT kit (Evrogen, Russia) and random primers (Random (dN)10 primer; Evrogen, Russia). This was followed by DNA amplification, fragmentation, and transcription of DNA with biotin-labeled nucleotides according to the protocols described above.

After completing the sample preparation stages for both DNA- and RNA-containing pathogens, the obtained biotin-labeled DNA was combined into a single sample and hybridized onto a microarray according to the manufacturer’s instructions (CustomArray, USA), followed by washing for reuse.

### Mathematical processing and analysis of hybridization signals

Primary hybridization signals in the form of ECD files were converted to CSV format using the Electra Sense Analysis 3.4.2 software (CustomArray, USA). Statistical processing of the hybridization signals was performed using the programming support environment R version 4.3.1 Beagle Scouts (GNU3 license [17]) and the RStudio shell version 2024.04.2 Chocolate Cosmos (GNU3 license [18]).

### Hybridization signals standardization

As a result of standardizing the obtained primary hybridization signals, standardized hybridization signals (SHS) were calculated in Z-units. This value characterizes the variability of the probe signal relative to the average signal of the negative control probe pool. The calculation was performed using the formula:

Z=(X−MNC)/SDNC,

where *Z* — standardized hybridization signals; *Х* — a primary hybridization signal of the probe; M_NC_ — arithmetic mean of the signals from negative control probes; SD_NС_ — standard deviation of the signals from negative control probes. SHS>3 Z was considered effective [19], SHS>4 Z as high, SHS>5 Z as very high, and SHS>10 Z was excluded from analysis, regarded as nonspecific/partial binding.

### Determination of optimal hybridization parameters

Optimal hybridization parameters were established through a series of experiments involving the hybridization of a model sample of total NA, pooled from two sources, one of which tested positive for the DNA- containing virus HAdV via PCR, while the other tested positive for the RNA-containing virus SARS-CoV-2. The pooled sample was divided into three aliquots, each undergoing a sample preparation cycle and then being united into a single sample. The sufficient quantity of biotin-labeled DNA for the experiment was obtained through this method.

Previously, during the development of a DNA microarray for the detection of bacterial CAP pathogens, we determined the optimal hybridization parameters for DNA on the microarray; they are the following: target DNA fragment size of 300 nt and the amount of hybridized DNA of 2 μg [20]. Given the known inverse relationship between the specificity and efficiency of DNA hybridization and hybridization temperature [21], the selection of hybridization temperatures (45, 47, and 49°C) was carried out using the established parameters. Each combination of hybridization parameters was tested six times, performing sequential DNA hybridizations on the microarray followed by washing. For each series of tests, we determined the hybridization efficiency, signal- to-noise ratio, and validity of hybridization.

Hybridization efficiency was assessed by calculating the percentage of effective probes with SHS≥3 Z from the total number of probes, excluding negative control probes, as well as the percentage of effective specific probes with SHS≥3 Z from the total number of specific probes. Specific probes were defined as those intended for the detection of HAdV and SARS-CoV-2 present in the model sample.

The signal-to-noise ratio was calculated as the ratio of the mean SHS of effective probes (SHS≥3 Z) to the threshold value of effective SHS (SHS=3 Z).

The validity of hybridization was evaluated as the ratio of the mean SHS of effective specific probes (SHS≥3 Z) to the mean SHS of effective nonspecific probes (SHS≥3 Z), excluding negative control probes. Specific probes were defined as those for the detection of HAdV and SARS-CoV-2, while probes for the detection of other viruses were considered nonspecific.

### Assessment of hybridization results reproducibility

To conduct this assessment, biotin-labeled DNA from the pooled sample containing HAdV DNA and SARS-CoV-2 RNA was used. The sample was hybridized on three different microarray slides and three times on a single microarray slide, followed by washing.

We calculated the paired Spearman’s rank correlation coefficients (ρ) between the hybridization signals of the probes obtained from hybridizations on the same slide, as well as the coefficient of variation (*Cv*) of the hybridization signals of the probes on different slides according to the formula:

Cv=(SD/M)⋅100%,

where *Cv* — the coefficient of variation, SD — the standard deviation of SHS probe, M — arithmetic mean of SHS probe.

### Determination of the significant signal threshold for the detection of viral community-acquired pneumonia pathogens

The significant signal threshold (SST) is defined as such SHS probe level, the excess of which is considered indicative of the presence of the corresponding CAP pathogen in the sample. The SST was established in such a way that the interpretation of DNA microarray hybridization results was comparable to those obtained using PCR analysis. We used clinical samples from patients with X-ray confirmed pneumonia, in which CAP pathogens were identified by PCR using the test systems AmpliSens ORVI-Screen-FL and AmpliSens CoV-Bat-FL (Central Research Institute of Epidemiology of Rospotrebnadzor, Russia). A total of selected samples containing DNA/RNA from viral CAP pathogens were selected for testing. The pathogens included HAdV (10 units), HBoV (8 units), SARS-CoV-2 (12 units), HPIV3 (10 units), HRSV (12 units), and RV (12 units).

After completing the DNA/RNA sample preparation protocol, each sample was hybridized on the DNA microarray according to the conditions established in the previous stage. Based on the obtained hybridization data, ROC analysis was conducted, calculating the sensitivity and specificity of the pathogen detection results for the tested SST set. When calculating, only effective probes (SHS≥3 Z) were taken into consideration.

For each sample, a ROC curve was plotted using a set of threshold SHS values under testing, ranging from 3 to 6 Z in increments of 0.1. For each threshold SHS value, specificity and sensitivity values were calculated using the formulas:

Sensitivity=TP/(TP+FN);Specificity=TN/(TN+FP),

where TP — the number of true-positive probes (designed for detecting a pathogen with SHS greater than the threshold value under test); FP — the number of false-positive probes (not designed for detecting a pathogen with SHS greater than the threshold value under test); TN — the number of true-negative probes (not designed for detecting a pathogen with SHS less than or equal to the threshold value); FN — the number of false-negative probes (designed for detecting a pathogen with SHS less than or equal to the threshold value).

Under the plotted ROC curves, the area under curve (AUC) was calculated, and threshold SHS values were determined at the point of maximum Youden’s index (the optimal sensitivity and specificity relationship) and at the point of maximum test specificity. The identified threshold SHS values were averaged for the set of samples containing a single pathogen. The obtained averaged threshold SHS value, corresponding to the point of maximum specificity, was considered the SST for the specific pathogen detection.

For all tested samples, the presence of nonspecific probes with SHS greater than the previously established SST was checked. Negative controls consisted of swab samples from the nasopharyngeal and oropharyngeal mucous membranes from nearly healthy donors who exhibited no clinical or laboratory CAP signs, whose PCR testing showed negative results (n=6).

### Optimal probe selection for specific detection of viral pathogens causing community-acquired pneumonia

Among all probes constituting the design of the DNA microarray, optimal specific probes were selected for the detection of each pathogen. For this purpose, during the hybridization of a sample set containing a specific pathogen, effective specific probes were identified, whose SHS exceeded SST of pathogenspecific probes established previously. The average SHS of each of these probes and its activity — the percentage of samples for which the probe SHS exceeded SST — were calculated. The optimal set of specific probes for detecting each pathogen was identified by maximizing two key parameters.

Using the BLASTN program of the BLAST+ 2.10.0 program package [22], the origin area for each selected probe was established — a fragment of the reference pathogen genome, to which the probe aligns with 100% identity. The reference genome fragments were annotated using the NCBI Nucleotide database [15].

### Statistical data processing

Calculations were made in the freely distributable programming environment R version 4.3.1 Beagle Scouts (GNU3 license [17]) using the RStudio shell version 2024.04.2 Chocolate Cosmos (GNU3 license) [18]. Descriptive statistics were presented with the median (Me), first and third quartiles [Q1 and Q3]. Reproducibility metrics were assessed using Spearman’s correlation coefficient (ρ) and the coefficient of variation (*Cv*). Sensitivity and specificity of the hybridization results were evaluated by plotting ROC curves to determine the AUC and Youden’s index. To compare random variables between two groups, the Mann–Whitney U test was used, with differences reported alongside the 95% confidence intervals. When conducting multiple comparisons, calculated levels of statistical significance were adjusted using the Benjamini–Hochberg procedure, with differences considered statistically significant at a corrected p-value of less than 0.05.

## Results

### DNA microarray design development for the indication of viral community-acquired pneumonia pathogens

During the DNA microarray design process, nucleotide sequences of DNA probes for the detection of adenovirus, bocavirus, respiratory syncytial virus, metapneumovirus, human parainfluenza virus, rhinovirus, and coronavirus were selected using our own software disprose. Local databases of target and non-specific nucleotide sequences were created using the NCBI Nucleotide and GISAID databases. Effective hybridization of DNA probes should occur for target sequences, while hybridization with non-target sequences is undesirable.

Nucleotide sequences from the reference annotated genomes of the main viral CAP pathogens (maternal sequences) were selected and divided into segments of established lengths, forming a pool of candidate probes. The following physicochemical parameters were used for further probe selection: length — 24–32 nt, guanine and cytosine content — 40–60%, the number of homogenous repetitions <5, minimal folding energy ≥0 kcal/mol, melting temperature — 55–60°C. The selected probes were aligned with the target and non-specific sequence databases using the BLAST algorithm. Then, there were selected specific probes with complete coverage of the target sequences (100% without gaps) and no cross-hybridization with non-target sequences (coverage <50%). The microarray design included 544 target DNA probes, each with a length of 25–30 nt. The probes were synthesized on slides.

### Selection of DNA hybridization temperature on microarrays

**Figure 1.**
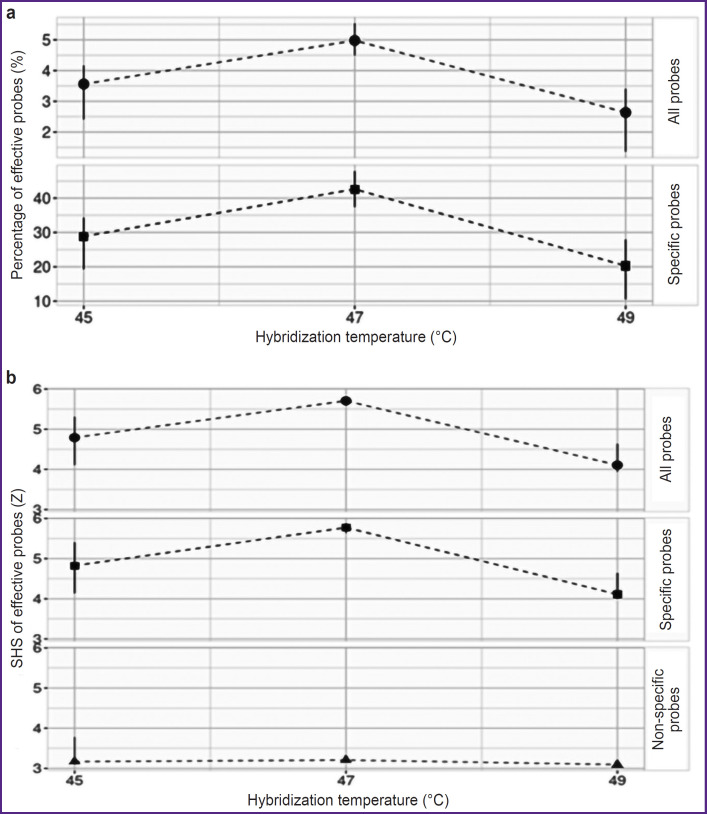
Hybridization efficiency indicators of DNA on a DNA microarray at different hybridization temperatures: (a) percentage of effective probes; (b) standardized hybridization signal (SHS) of effective probes. The graphs were constructed using values of the median, the first and third quartiles

Following sample preparation, the pooled sample — confirmed by PCR to contain HAdV DNA and SARS-CoV-2 RNA — was hybridized on the microarray, and the selection of various hybridization temperature regimes (45, 47, or 49°C) was conducted. Probes for the HAdV detection identified only the HAdVB variant; thus, specific probes referred only to those intended for the HAdVB and SARS-CoV-2 detection. The optimal hybridization temperature, at which the highest percentage of effective probes and the highest SHS values of effective probes were recorded, was 47°C (Figure 1).

### Assessment of the reproducibility of hybridization results

The developed DNA microarray demonstrated high hybridization signal reproducibility. The paired correlation coefficients of hybridization signals between different microarrays, as well as when reusing the same microarray, were above 0.90.

### Determination of the significant signal threshold for the detection of viral pathogens causing community-acquired pneumonia

**Figure 2.**
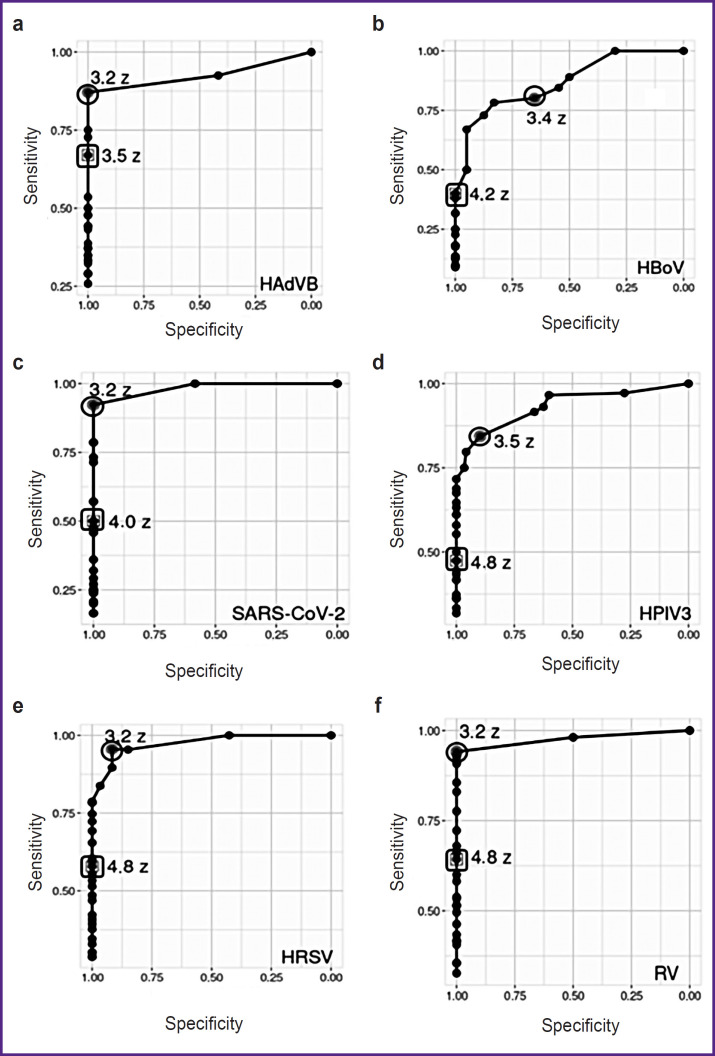
ROC curves for the detection of viral community- acquired pneumonia pathogens from clinical samples: (a) HAdVB; (b) HBoV; (c) SARS-CoV-2; (d) HPIV3; (e) HRSV; (f) RV. There are displayed the averaged ROC curves plotted based on the median values of specificity and sensitivity for a set of samples containing pathogen DNA/ RNA. The circle indicates the point of the standardized hybridization signal (SHS) with the maximal Youden’s index, while the square marks the SHS point with the highest test specificity, corresponding to the significant signal threshold (SST)

For six viral CAP pathogens (HAdVB, HBoV, HPIV3, HRSV, RV, and SARS-CoV-2), the ranges of hybridization signals from specific probes were analyzed using ROC analysis, and the probe SSTs were determined, with signal values exceeding the threshold interpreted as the presence of pathogen DNA/RNA in the sample. For each pathogen, the average threshold values exceeded the average values of the Youden’s index (J): for HAdVB — SST=3.5 Z, J=3.2 Z; for HBoV — SST=4.2 Z, J=3.4 Z; for SARS-CoV-2 — SST=4.0 Z, J=3.2 Z; for HPIV3 — SST=4.8 Z, J=3.5 Z; for HRSV — SST=4.8 Z, J=3.2 Z; for RV — SST=4.8 Z, J=3.2 Z (Figure 2).

### Optimal probe selection for specific detection of viral pathogens causing community-acquired pneumonia

As a result of testing, probes with the highest average SHS and maximal activity were selected for each pathogen (HAdVB, HBoV, HPIV3, HRSV, RV, and SARS-CoV-2), which we defined as optimal for specific pathogen detection using DNA microarray. Analysis of the origin areas of the selected probes indicated that most of them originated from the coding regions of viral genomes or the non-coding region of the 5’-UTR (see the Table).

**Table:** Optimal DNA probe kits for specific detection of viral pathogens causing community-acquired pneumonia

Probe ID	Probe nucleotide sequence	Percentage composition of guanine and cytosine	Melting temperature (°С)	Activity (%)	Standardized hybridization signal, Me [Q1; Q3]	Reference sequence	Characteristics of the reference sequence region
***Human adenovirus B (HAdVB)***							
12079	attgcatgaaagcctttgctgtctt	40	56.9	70	7.0 [5.7; 7.9]	NC_011202	Gene ID: 24271507. Coding product: membrane glycoprotein E3 precursor
5472	ttgcatgaaagcctttgctgtctt	42	56.7	50	5.6 [5.2; 6.8]		
5470	gattgcatgaaagcctttgctgtc	46	56.3	40	7.4 [6.4; 8.2]		
15817	aagatactgagcagtctccgatgttg	46	57.5	60	4.4 [4.3; 4.5]	NC_011202	Gene ID: 24271518. Coding product: core protein V precursor
3143	gatactgagcagtctccgatgttg	50	56.2	50	6.0 [4.1; 7.7]		
9999	gatactgagcagtctccgatgttgt	48	57.4	50	5.3 [4.2; 5.8]		
10001	tactgagcagtctccgatgttgtgt	48	58.7	40	5.8 [4.6; 7.2]		
15819	gatactgagcagtctccgatgttgtg	50	58.3	40	4.7 [4.2; 5.3]		
5150	ctggaggaagacagtttggaggag	54	58.4	40	4.3 [3.5; 5.9]	NC_011202	Region 24188–26348 nt. Non-coding intron
5152	gaggaagacagtttggaggaggaa	50	57.8	40	3.8 [3.7; 3.8]		
***Human bocavirus (HBoV)***							
661	tgggtgcttcctggttataaatac	42	56.2	75	5.0 [4.4; 5.1]	FJ170280	Gene ID: 7768237. Coding product: nonstructural protein NP1
1749	gtgggtgcttcctggttataaatac	44	57.2	38	4.4 [4.4; 5.4]		
1109	accacatcctgaagatgatcctgtc	48	57.6	50	4.3 [4.3; 4.6]	FJ170280	Gene ID: 7768240. Coding product: nonstructural protein NS1
54	gcaccacatcctgaagatgatcct	50	58.0	50	5.1 [4.4; 5.8]		
1111	cacatcctgaagatgatcctgtcag	48	56.0	38	4.7 [4.7; 4.9]		
56	accacatcctgaagatgatcctgt	46	56.8	38	4.7 [4.4; 4.9]		
348	gggtcctttgtcctactcattcac	50	57.5	38	5.4 [4.9; 6.0]	NC_012564	
347	ggggtcctttgtcctactcattca	50	59.0	25	4.9 [4.6; 5.2]		
1250	ctaccacgcaaccctagataacgaa	48	59.4	25	4.8 [4.7; 4.9]		
***Human coronavirus SARS-CoV-2 (SARS-CoV-2)***							
733	gtaatgttcaactcagggttattggac	41	56.7	92	5.3 [4.9; 5.7]	EPI_ISL_481284	Region 1–264 nt 5’-UTR
229	tctggtaaagttgagggttgtatgg	44	57.5	50	4.8 [4.5; 6.3]		
19	tggtacaactacacttaacggtct	42	56.0	50	5.3 [4.7; 5.9]		
557	catgcagatcaacttactcctacttgg	44	57.4	50	5.6 [5.1; 7.0]	EPI_ISL_661270	
404	ctcctacttggcgtgtttattctaca	42	57.3	33	5.5 [5.1; 6.2]		
630	tttgctgctgcttgacagattgaac	44	57.0	50	5.0 [4.6; 6.3]	EPIISL661270	Gene ID: 1489680. Coding product: ORF1a polyprotein (1-180 aa), nonstructural protein Nsp1
260	tttgctgctgcttgacagattgaa	42	56.0	42	5.1 [4.3; 5.2]		
601	ctagtcaatccatcattgcctacacta	41	56.7	33	5.8 [4.7; 7.1]		
358	gctatgaggcccaatttcactatta	40	56.0	50	4.8 [4.3; 5.1]	EPI_ISL_481284	
15793	tggcctcttattgtaacagctttaagg	41	58.2	33	5.9 [5.2; 6.8]	EPI_ISL_402124	Gene ID: 1489680. Coding product: ORF1a polyprotein (4118-4230 aa), Nsp9 protein
***Human parainfluenza virus 3 (HPIV3)***							
171	ggagtggcagttgtacaaaacaga	46	57.0	100	5.9 [5.8; 6.8]	KY369866	Gene ID: 911955. Coding product: HPIV3gp1 nucleocapsid protein
10386	caatgagatcactagttgcagtcatcaac	41	57.0	100	5.9 [5.3; 6.6]	KY369866	Gene ID: 911956. Coding product: phosphoprotein HPIV3gp2
4748	aggaaggatacagaagagagcaatcg	46	57.5	90	5.5 [5.5; 7.4]	NC_001796	Gene ID: 911958. Coding product: D-protein HPIV3gp3
8556	gaaaggaaggatacagaagagagcaatc	43	56.8	80	7.3 [7.1; 7.6]		
6678	aaggaaggatacagaagagagcaatcg	44	57.8	80	6.4 [5.8; 7.0]		
2504	ggaaggatacagaagagagcaatcg	48	56.6	70	6.6 [5.7; 6.6]		
2240	aaatcaactaatatctcctcggccc	44	57.5	80	7.8 [7.0; 8.4]	NC_001796	Gene ID: 911959. Coding product: C-protein HPIV3gp4
4517	taaatcaactaatatctcctcggccc	42	57.6	80	6.2 [6.1; 6.8]		
6436	ataaatcaactaatatctcctcggccc	41	57.8	70	7.3 [6.8; 7.3]		
***Human respiratory syncytial virus (HRSV)***							
2938	tatgtcacgaaggaatccttgcaaa	40	56.5	83	6.8 [6.1; 7.3]	AY911262	Region 1–44 nt 5-UTR
1108	atgtcacgaaggaatccttgcaaa	42	56.3	75	6.2 [5.0; 6.6]	FJ170280	
1256	gcaacatcctccatcatggttaat	42	55.5	100	6.7 [6.0; 7.7]	NC_038235	Gene ID: 1489827. Coding product: HRSVgp10 polymerase
1254	atgcaacatcctccatcatggtta	42	56.4	100	5.7 [5.2; 6.5]		
4708	gcaacatcctccatcatggttaatac	42	56.7	83	6.5 [6.3; 7.2]		
5182	tgccactcaacaatttctccaacatc	42	57.3	75	5.4 [5.1; 6.2]	KU950500	Gene ID: 1489826. Coding product: protein M2-2
6927	gatgccactcaacaatttctccaacat	41	57.5	67	7.8 [7.0; 8.2]		
6928	atgccactcaacaatttctccaacatc	41	57.5	75	5.2 [4.8; 6.8]		
2939	atgtcacgaaggaatccttgcaaat	40	56.5	75	5.5 [5.1; 6.5]	AY911262	
1109	tgtcacgaaggaatccttgcaaat	42	56.3	75	6.7 [5.5; 7.2]	FJ170280	
***Human rhinovirus (RV)***							
175	ccaaattacggacaaggtgtgaaga	44	57.5	100	5.9 [5.0; 7.2]	EU096002	Region 1–603 nt 5’-UTR
59	tctttgagcgttcgatcaggtgaa	46	57.1	100	7.4 [5.2; 8.0]		
78	aattacggacaaggtgtgaagagc	46	56.9	100	6.1 [4.7; 6.8]		
76	ccaaattacggacaaggtgtgaag	46	56.5	100	5.9 [5.2; 7.7]		
285	ttccgctatttcccatagtagacctg	46	59.3	92	6.5 [5.3; 7.4]		
225	atgctttcgaagtcatttggttggtc	42	57.6	83	6.4 [6.0; 8.2]		
61	attctggaaaattcttggctggtc	42	55.3	100	6.1 [5.2; 8.1]	EU096000	Region 1–576 nt 5’-UTR
58	tgcctacacagaacttagtaccat	42	55.8	100	5.9 [5.0; 7.4]		
93	cgctatttcccatagtagacctgg	50	58.0	83	6.9 [6.1; 8.4]	EU096008	Region 1–557 nt 5’-UTR

## Discussion

At the first stage of developing the DNA microarray using the GenBank and GISAID databases, along with our own disprose program, DNA probes were selected to indicate the primary viral CAP pathogens currently circulating globally. A total of 544 DNA probes were synthesized on slides. Then, the hybridization temperature of the pooled sample containing HAdVB DNA and SARS-CoV-2 RNA was tested on the developed microarray. At the optimal hybridization temperature of 47°C, the highest percentages of effective probes and the highest SHS values of effective probes were recorded (see Figure 1). This selected hybridization temperature coincided with the optimal hybridization temperature for a previously developed microarray aimed at detecting bacterial CAP pathogens [20]. This indicates that the development of a universal microarray for the detection of both bacterial and viral CAP pathogens with unified DNA hybridization parameters may be feasible in the future. It should be noted that the introduction of an additional reverse transcription step in the sample preparation protocol for RNA-containing CAP pathogens does not affect the hybridization efficiency.

The paired correlation coefficients of hybridization signals between different microarrays and when reusing a single microarray corresponded to the recommended in literature value of at least 0.90 [23]. This suggests the potential for reusing the developed DNA microarrays without loss of hybridization quality, thereby reducing the cost of the analysis.

For six viral CAP pathogens (HAdVB, HBoV, HPIV3, HRSV, RV, and SARS-CoV-2), ROC analysis for determining SST showed that on the averaged ROC curves (see Figure 2) for all pathogens the SHS was higher at the SST point than at the Youden’s index point (the optimal sensitivity and specificity relationship). The DNA microarray sensitivity was arbitrarily reduced to filter out some signals from effective specific probes in cases of possible asymptomatic pathogen carriage. According to literature data [7, 8], respiratory viruses, including those causing CAP, can be detected using molecular genetic methods in clinically healthy individuals without symptoms of disease; however, the amount of pathogen DNA/RNA during carriage is lower than those during active infection. The probability of pathogen NA specific hybridization on the DNA microarray during carriage is lower than during active infection, and effective hybridization during carriage will only be detected with single spot probes (the microarray surfaces where the molecules of a single probe are synthesized). Although the target molecule concentration does not affect the hybridization strength of a single probe molecule [24], it determines the proportion of reacted spot probe molecules, the cumulative hybridization signal of which is interpreted as the hybridization signal of the corresponding probe [25]. As a result, the low concentration of pathogen DNA/RNA in samples in cases of carriage leads to incomplete hybridization of the spot probes and a reduced hybridization signal level. This allows us to differentiate between specific probe SHS values during carriage and those during active infection.

Thus, overestimating the SST value enables the exclusion of nonspecific hybridization signals and specific probes binding signals in cases of healthy carriage from the interpretation of DNA microarray hybridization results. The associated loss of sensitivity can be considered acceptable because the final hybridization results interpretation is qualitative (detected — not detected) and does not depend on the number of effective specific probes exceeding the SST. The differences in SST values for different pathogens confirm that the SST value is not universal for all pathogens detected by the microarray, which requires individual SST calculations for each CAP pathogen under test.

For the detection of each pathogen (HAdVB, HBoV, HPIV3, HRSV, RV, and SARS-CoV-2), we selected a set of probes with maximal average SHS and maximal activity (see the Table). Most selected probes did not exhibit 100% activity; positive signals were observed from different sets of specific probes during the hybridization of different samples containing DNA/RNA from a single pathogen. According to literature data [24], various DNA probes specific to the same molecular target, and even to the same target region, differ in affinity and, consequently, in the hybridization signal level. During the hybridization of different samples, the signal level of each specific probe is variable and may not always be definitively interpreted as positive. This justifies the use of a pool of specific probes in the DNA microarray design. Most of the chosen DNA probes originated from coding regions of the viral genome or the 5’-UTR region. The probe sequences were selected to specifically interact with the viral genetic sequences regardless of strain, gene variant, and other individual characteristics [14]. Therefore, the probe origin areas can be considered as conserved areas of the viral genomes suitable for developing molecular diagnostic tests, but not appropriate for typing assays of CAP pathogens.

Thus, the DNA microarray provides effective indication of viral CAP pathogens, when each pathogen is detected by a set of specific probes. The application of significant signal thresholds for the probes allows differentiation between clinically significant infection and CAP pathogens carriage. The use of random primers ensures the unification of sample preparation and reduces labor costs. The DNA microarray is suitable for multiple uses, which also lowers the analysis cost.

## Conclusion

While developing the DNA microarray for the indication of viral pathogens causing community- acquired pneumonia, DNA probes were selected and synthesized on slides for the detection of adenovirus, bocavirus, metapneumovirus, human parainfluenza virus, rhinovirus, and coronavirus. The optimal DNA hybridization temperature was established at 47°C, at which quite effective, specific, and reproducible hybridization signals were detected. Threshold values for significant probe signals were calculated for the specific detection of the selected viruses, allowing the interpretation of hybridization results to be identical to those obtained from PCR analysis. There was determined a list of probes for the specific detection of these viruses, characterized by effective hybridization signals. The developed DNA microarray can serve as a modern tool for laboratory diagnostics and monitoring of relevant viral CAP pathogens.
